# Efficient deep learning models for oral squamous cell carcinoma classification in histopathological images

**DOI:** 10.1038/s41598-026-44424-5

**Published:** 2026-05-12

**Authors:** Jatender Kumar, Munish Kumar, M. K. Jindal

**Affiliations:** 1 Department of Computer Science and Application, Panjab University, Chandigarh, India; 2 Department of Computer Science, SGGS College, Chandigarh, India; 3 Department of Computational Sciences, Maharaja Ranjit Singh Punjab Technical University, Bathinda, Punjab India; 4 Department of Computer Science and Applications, Panjab University Regional Centre, Muktsar, India

**Keywords:** OSCC, Histopathology, Image classification, Deep learning, Cancer, Computational biology and bioinformatics, Mathematics and computing

## Abstract

Recent advances in deep learning have significantly improved the accuracy and efficiency of disease classification in digital pathology. Early diagnosis and precise classification of histopathological images are crucial for enabling timely treatment and improving therapeutic outcomes. Oral squamous cell carcinoma (OSCC) is one of the most common malignancies in the oral cavity, with manual histopathological examination serving as the gold standard for diagnosis—though it is time-consuming and subject to observer variability. This study investigates the performance of four deep learning convolutional neural network (CNN) models—ResNet50 (residual blocks), DenseNet201 (dense connectivity), EfficientNetB0 (compound scaling), and ConvNeXt_Tiny (transformer-based convolutions)—for binary classification (benign vs. carcinoma) of 10,000 histopathological images. Among the models, EfficientNetB0 achieved the highest accuracy of 97.6% and an ROC-AUC score of 0.9963, demonstrating superior generalization and discriminative power. ConvNeXt_Tiny followed with an accuracy of 95.92%, DenseNet201 with 86.08%, and ResNet50 with the lowest accuracy of 71.52%. The comparative analysis underscores the advantages of modern CNN architectures over traditional residual networks, supporting the integration of deep learning models into diagnostic frameworks for improved detection of oral squamous cell carcinoma.

## Introduction

Oral cancer is a significant global public health concern, particularly in developing countries like India, where it imposes a substantial burden on both the healthcare system and the patient population. This paper focuses on classifying oral histopathology images into benign and carcinoma categories. Examples of benign lesions are fibroma (connective tissue lesion), papilloma (epithelial wart-like lesion), and hyperkeratosis (thickened keratinized mucosa). Carcinoma lesions are diagnosed as squamous cell carcinoma (malignant epithelial tumor) or verrucous carcinoma (apparent slow-growing, well-differentiated version of squamous carcinoma). Squamous cell carcinoma (SCC) is a malignant epithelial tumor originating from the stratified squamous epithelium of the oral mucosa. It is the most frequent form of oral malignancy, usually involving the tongue, floor of the mouth, and buccal mucosa. Among all types of oral cancers, oral squamous cell carcinoma (OSCC) is the most prevalent, accounting for over 90% of cases^1^. In India, oral cancer represents nearly 40% of all cancer cases, with an estimated 60,000 new diagnoses annually and more than five deaths occurring every hour. Notably, OSCC tends to affect a younger demographic in India compared to Western countries^2^. Between 1990 and 2021, India witnessed an increase in mortality rate (from 5.32 to 5.92), incidence (10.1 to 10.15), prevalence (15.71 to 25.46), and disability-adjusted life years (DALYs) (152.94 to 163.61) per 100,000 populations^3^. Males are disproportionately affected, accounting for 76.7% of cases in 2018^4^. This life-threatening malignancy originates from the epithelial lining of the oral cavity and is strongly associated with several risk factors, including tobacco use in all forms, alcohol consumption, betel quid chewing, and areca nut usage—a known carcinogen. These habits are widespread in South and East Asia and contribute to poor oral hygiene. Symptoms of oral cancer include difficulty eating and speaking, mouth irritation, and visible facial lesions. Unfortunately, these signs are often nonspecific and easily overlooked, leading to late-stage diagnosis in many patients^5^.

Early detection of oral squamous cell carcinoma (OSCC) is critical for successful treatment, yet it remains a significant challenge, especially in resource-constrained settings. While traditional pathology plays a crucial and highly specialized role in clinical diagnostics, it has notable limitations. Histopathological examination through tissue biopsy is the standard approach; however, it is invasive, time-consuming, and subject to inter-observer variability, often leading to diagnostic delays. Manual inspection of histopathological images requires substantial time and expertise, and its subjective nature can result in inconsistent interpretations. These limitations highlight the urgent need for automated and scalable diagnostic solutions. The advancement of artificial intelligence (AI), particularly deep learning, offers promising avenues to address these challenges by enhancing diagnostic accuracy and efficiency. The integration of deep learning into histopathological image analysis has rapidly advanced the field of computational pathology. Recent breakthroughs in deep learning have enabled new possibilities for digital pathology, especially with histopathological images that are high-resolution gigapixel images containing fine-grained tissue morphology across multiple structures. These images provide rich diagnostic information but also present significant technical challenges due to their size, complexity, and the need for patch-based analysis. To address these issues, the development of scalable diagnostic tools is essential for early detection, reducing the healthcare burden, and improving prognosis. This study contributes to the growing body of research by evaluating the classification performance of histopathological images in OSCC using state-of-the-art deep learning models. Four convolutional neural network (CNN) architectures—ResNet50, DenseNet201, EfficientNetB0, and ConvNeXt_Tiny—were selected for comparison, each representing distinct architectural principles and computational strategies. The aim is to investigate how modern innovations such as transformer-inspired convolutional structures and compound scaling influence classification performance in real-world histopathological tasks. The primary objective is to identify the most effective architectural paradigm for binary classification and to offer practical insights into the trade-offs between model complexity, accuracy, and inference reliability.

This paper is organized into seven main sections. The introduction provides background information relevant to the study. Section “Related work”, reviews existing literature to contextualize the research. Section “Materials and methods”, details the dataset used, the patch generation process, training and classification techniques, and the evaluation metrics employed. Section “Experimental results and analysis”, presents various performance assessments, including overall performance metrics, model accuracy curves, per-class and macro-averaged metrics, precision-recall (PR) curves, probability- and threshold-based metrics such as the receiver operating characteristic (ROC) curve, training and validation loss curves, and the confusion matrix. Section “Comparative analysis” within four models. Section “Discussion” highlights the comparison of proposed work with previous studies and the practical implications of the findings. Finally, Section “Conclusion and future work” concludes the paper with a summary and outlines direction for future work, followed by a comprehensive list of references.

### Background

Convolutional Neural Network (CNN) models have become transformative technologies in digital pathology and medical imaging, enabling automatic, hierarchical feature learning directly from histopathological images. Models such as AlexNet^6^, Xception^7^, VGG^8^, Inception^9^, and MobileNet^10^ have outperformed traditional approaches that rely heavily on handcrafted features. These models have contributed significantly to enhancing feature representation, improving generalization across datasets, and increasing computational efficiency. In this study, we focus on four of the most widely researched CNN models—ResNet50^11^, DenseNet201^12^, EfficientNetB0^13^, and ConvNeXt_Tiny^14^—to evaluate their performance in the classification of histopathological images. A brief description of these CNN architectures is provided in Table 1.

**Table 1:** CNN models.

Model	Year	Architecture	Parameters	Application
ResNet50 ^15^	2015	CNN with residual blocks	25.6 M	General purpose
DenseNet201 (Haung et al. 2017^31^)	2017	CNN with dense connections	20 M	General purpose with strong feature reuse
EfficientNetB0 ^16^	2019	CNN with compound scaling	5.3 M	Mobile friendly/edge devices
ConvNeXt_Tiny ^17^	2022	Modernized CNN inspired by Transformers	28.6 M	Advanced tasks with better generalization

ResNet50 (Residual Networks) is a widely used CNN model based on the concept of residual learning, introducing skip connections to address the vanishing gradient problem in deep networks. With approximately 25.6 million parameters, it offers moderate inference speed while delivering a strong baseline performance in image classification tasks. Its architecture enables the construction of significantly deeper networks without performance degradation and has served as the foundation for many subsequent CNN architectures. DenseNet201 (Densely Connected Networks) is another prominent CNN model characterized by dense connections between layers. Each layer receives input from all preceding layers, promoting efficient feature reuse and improved gradient propagation. This architecture enhances information flow throughout the network and facilitates learning. With around 20 million parameters, DenseNet201 strikes a balance between model complexity and performance, making it well-suited for general image classification tasks. EfficientNetB0 employs compound scaling to uniformly scale network depth, width, and input resolution using a set of fixed scaling coefficients. As the baseline model in the EfficientNet family, it is highly efficient and lightweight, with only 5.3 million parameters. Despite its compact size, it achieves excellent accuracy and is particularly suitable for deployment in resource-constrained environments due to its fast inference and minimal computational requirements. ConvNeXt_Tiny represents a modern class of CNNs inspired by transformer architectures. While maintaining a convolutional backbone, it incorporates design principles from Vision Transformers (ViT), such as long-range dependencies, depthwise separable convolutions, Gaussian Error Linear Unit (GELU) activations, and advanced training strategies. With 28.6 million parameters, the Tiny variant achieves a favourable trade-off between accuracy and inference speed. These enhancements enable ConvNeXt to effectively bridge the gap between the efficiency of traditional CNNs and the representational power of transformer-based models.

## Related work

For the diagnosis and classification of oral squamous cell carcinoma (OSCC), numerous studies have explored the potential of transfer learning and ensemble methods to enhance model performance, particularly in data-limited settings. Research by Rahman et al.^18^, Panigrahi et al.^19^, and Saikia et al.^20^ has demonstrated the effectiveness of transfer learning using pre-trained CNN models such as AlexNet, VGG16, VGG19, InceptionV3, ResNet50, and MobileNet. When fine-tuned on domain-specific datasets, these models have achieved high classification accuracies, with ResNet50 frequently outperforming others, exceeding 96% accuracy in multiple studies. To further mitigate data scarcity and enhance diagnostic precision, researchers such as Das et al.^21^, Deo et al.^22^, and Saikia et al.^20^ have proposed ensemble learning frameworks that leverage the strengths of multiple CNN models. These ensemble approaches generally outperformed individual models. For example, in Deo et al.^22^, a 2D Empirical Wavelet Transform (EWT)-based ensemble achieved 92% accuracy, surpassing the individual performance of ResNet50 and DenseNet201. Additionally, hybrid feature-based approaches have gained traction. Ahmad et al.^23^ combined deep CNN features with handcrafted descriptors such as GLCM, LBP, and HOG, and used an SVM classifier for final prediction. This hybrid method achieved robust diagnostic performance, reporting 97% accuracy and a 96.8% AUC.

Similarly, Das et al.^24^ combined CNN-based segmentation with Gabor texture filters and Random Forest classifiers to detect keratin pearls, a key histological feature of OSCC, achieving an accuracy of 96.88%. Complementing these CNN-based approaches, transformer models and few-shot learning techniques have recently been explored to further enhance generalizability and contextual understanding. Maia et al*.*^10^ conducted a comparative analysis of CNN and transformer-based models—including CoaTLite, PiT, and Vision Transformer (ViT)—using 3763 histopathological image patches from the NDB UFES dataset. While CNN models such as DenseNet121 (91.91%) and MobileNet (91.49%) demonstrated competitive performance, transformer models like ViT achieved slightly lower but still promising results (ViT Small: 90.42%), highlighting their potential with further fine-tuning and larger datasets. Beyond image classification, studies by Yang et al.^25^ and Silva et al.^26^ investigated diagnostic support tools aimed not only at classification but also at improving pathologist efficiency and grading precancerous lesions. Yang et al.^25^ demonstrated that deep learning models could reduce diagnostic time while increasing accuracy for both junior and senior pathologists. Silva et al.^26^ introduced a two-stage pipeline combining Mask R-CNN for nuclei segmentation with polynomial classification for dysplasia grading, achieving up to 97% AUC. Extending the scope to photographic image-based detection, Dutta et al.^27^ utilized Radial Basis Function Networks (RBFN) with feature descriptors such as SIFT and HOG, achieving an impressive accuracy of 99.99% with their RBFN-SDC model.

Although deep learning has significantly advanced oral cancer detection, most studies have focused on traditional CNNs or hybrid models. Limited research has compared newer architectures such as residual networks, dense networks, transformer-based models, and compound-scaled CNNs in this domain. These advanced models offer improved feature learning, efficiency, and scalability; however, their effectiveness in histopathological image analysis—especially in resource-limited settings—remains underexplored. This study addresses this gap by systematically evaluating and benchmarking these state-of-the-art models for accurate and robust oral cancer classification.

## Materials and methods

**Fig. 1:**
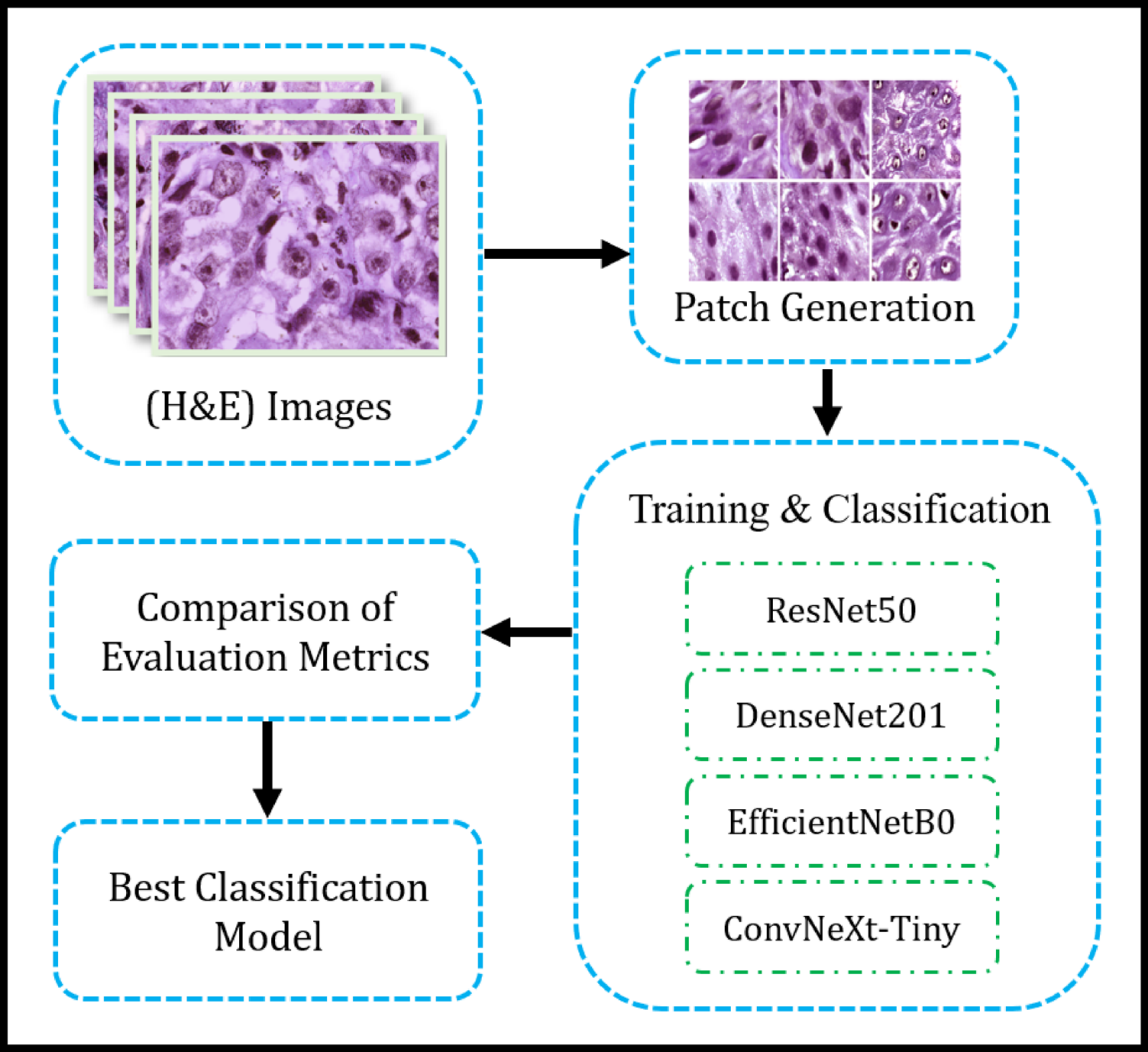
Process flow of oral cancer histopathology image classification.

The proposed approach in this paper consists of several stages, as illustrated in Fig. 1. The process flow is fine-tuned for high-resolution histological data to ensure that each stage is stable, reproducible, and robust to variations.

### Dataset

Oral benign and carcinoma histopathological images stained with hematoxylin and eosin (H&E) were collected from the ORCHID dataset^28^. These images serve as the primary input for classification. The dataset is publicly available on Zenodo under a CC-BY 4.0 license^29^. It contains histopathological images from 150 patients organized into different class folders. For this study, we selected the normal and OSCC class folders, which contain images at × 1000 magnification. The normal class folder consists of benign images.

In this paper, we used a total of 3000 images divided into two classes, with 1500 images per class for evaluation, as shown in Table 2. The study utilized 10,000 image patches generated from 5000 normal and 5000 OSCC histopathological images.

**Table 2:** Dataset of histopathological image and patches.

Class	Images (1000x)	Patches (64)
Normal	1500	5000
OSCC	1500	5000
Total	3000	10,000

### Patch generation

**Fig. 2:**
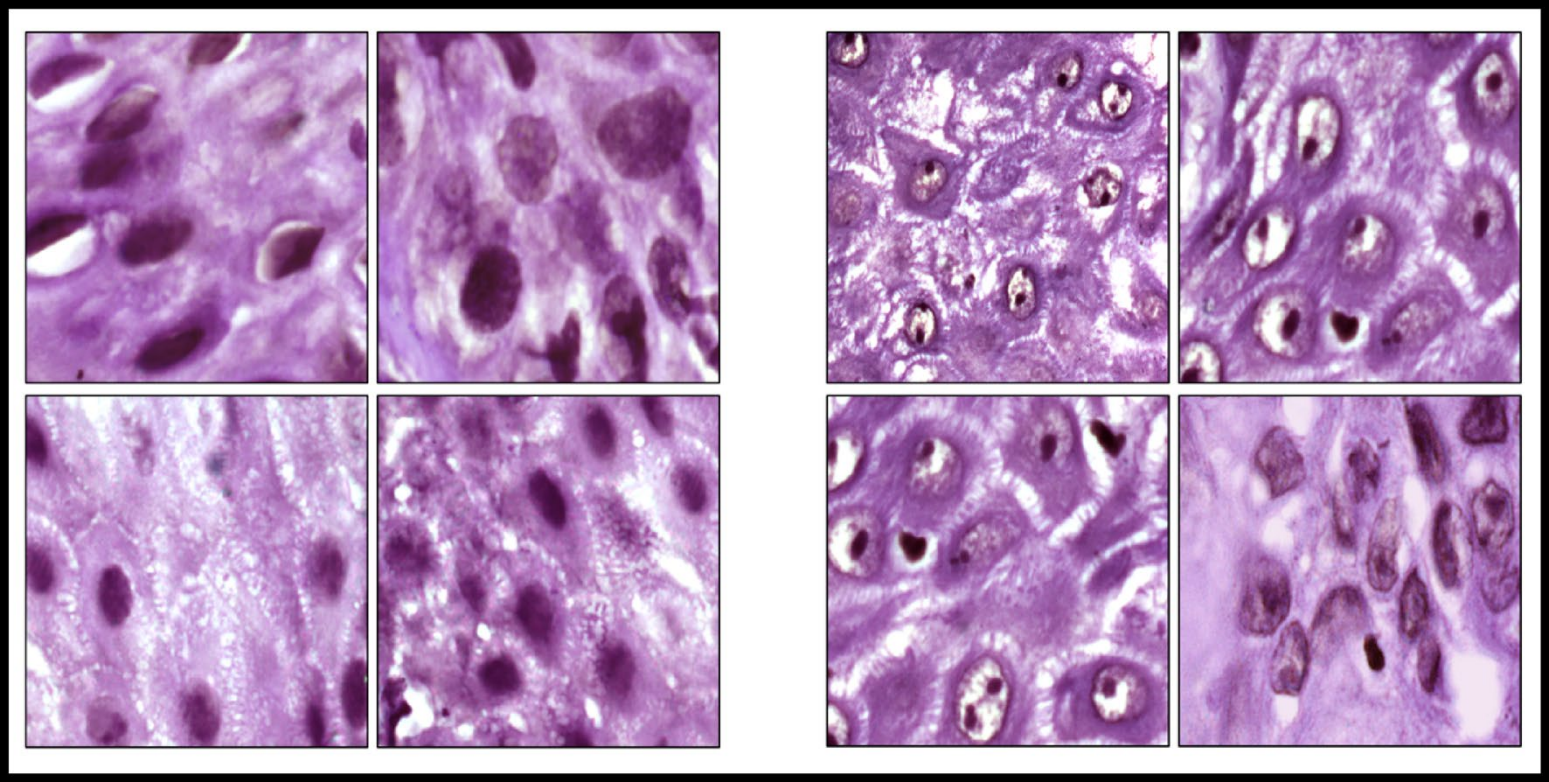
(left) Oral Benign images, (right) Oral squamous cell carcinoma.

To enhance training and capture fine-grained details, the images were downsampled into patches. This reduces the input dimensions significantly while enabling the model to learn localized tissue patterns. Overlapping patches of size 256 × 256 pixels were generated from 1000 × magnified histopathological images using Scikit-learn’s Patch Extractor^30^. Given the high resolution of the images, dividing each into fixed-size overlapping patches ensures computational feasibility and supports efficient model training. All extracted patches were then resized to 64 × 64 pixels to improve computational efficiency, standardize input dimensions, and minimize processing overhead during training. Examples of patches from oral benign and oral squamous cell carcinoma tissues are shown in Fig. 2.

### Training and classification

Four pretrained models—ResNet50, DenseNet201, EfficientNetB0, and ConvNeXt_Tiny—were used for training and classification based on ImageNet weights and transfer learning. All models were initialized with ImageNet pretrained weights. To fine-tune the models, all layers except the last five were frozen, allowing the final layers to adapt to the new dataset while retaining the pretrained features in the earlier layers. This approach balances preserving generalization with adapting to domain-specific features. The last fully connected layer was replaced with a two-output layer for binary classification. For consistency and fair comparison, identical hyperparameters were applied to all models: Adam optimizer, a base learning rate of 0.001, weight decay of 0.0005, and batch size of 64. Model training was optimized using mean squared error (MSE) loss and specific callbacks to improve efficiency. To prevent overfitting, early stopping was implemented, halting training if validation loss did not improve for 12 consecutive epochs. Cyclical learning rates were used with a minimum threshold of 1e-6. Additionally, if validation loss plateaued for 6 epochs, the learning rate was automatically reduced by a factor of 0.2. Model checkpointing saved the best weights based on validation accuracy, ensuring retention of the best-performing model version.

### Evaluation metrics

A comprehensive set of benchmark classification performance metrics was used in this study to evaluate the effectiveness of the proposed model. These include Matthews Correlation Coefficient (MCC), a robust metric that accounts for true and false positives and negatives. Balanced accuracy addresses imbalanced class distributions by averaging recall across classes. Accuracy measures the overall correctness of the model. The Jaccard Index is used to assess the similarity between actual and predicted labels. Sensitivity and specificity for both the benign and carcinoma classes provide class-wise evaluation, offering insights into the model’s detection capability for each class. To ensure equal weighting of all classes regardless of their size, macro-averaged sensitivity (recall) and specificity are computed. Log Loss quantifies the uncertainty of incorrect predictions, while the Receiver Operating Characteristic (ROC) score evaluates probabilistic predictions by measuring the trade-off between sensitivity and specificity. Together, these metrics provide a comprehensive assessment of the model’s performance.

1 $${\mathrm{Accuracy}} = \frac{{{\mathrm{TP}} + {\mathrm{TN}}}}{{{\mathrm{TP}} + {\mathrm{TN}} + {\mathrm{FP}} + {\mathrm{FN}}}}$$

2 $${\text{Balanced Accuracy}} = \frac{1}{2}\left. {\left( {{ }\frac{{{\mathrm{TP}}}}{{{\mathrm{TP}} + {\mathrm{FN}}}}{ }} \right. + { }\frac{{{\mathrm{TN}}}}{{{\mathrm{TN}} + {\mathrm{FP}}}}{ }} \right)^{{ }}$$

3 $${\text{Matthews Correlation Coefficient }} = \frac{{{\mathrm{TP}}.{\mathrm{TN}} - {\mathrm{FP}}.{\mathrm{FN}}}}{{\sqrt {\left( {{\mathrm{TP}} + {\mathrm{FP}}} \right)\left( {{\mathrm{TP}} + {\mathrm{FN}}} \right)\left( {{\mathrm{TN}} + {\mathrm{FP}}} \right)\left( {{\mathrm{TN}} + {\mathrm{FN}}} \right)} }}$$

4 $${\text{Jaccard Index}} = \frac{{{\mathrm{TP}}}}{{{\mathrm{TP}} + {\mathrm{FP}} + {\mathrm{FN}}}}$$

5 $${\mathrm{Sensitivity}} = { }\frac{{{\mathrm{TP}}}}{{{\mathrm{TP}} + {\mathrm{FN}}}}{ }$$

6 $${\mathrm{Specificity}} = { }\frac{{{\mathrm{TN}}}}{{{\mathrm{TN}} + {\mathrm{FP}}}}{ }$$

7 $${\mathrm{Macro}}\;{\text{Sensitivity }}\left( {{\mathrm{Macro}}\;{\mathrm{Recall}}} \right) = { }\frac{1}{{\mathrm{N}}}\mathop \sum \limits_{{{\mathrm{i}} = 1}}^{{\mathrm{N}}} \frac{{{\mathrm{TP}}}}{{{\mathrm{TP}}_{{\mathrm{i}}} + {\mathrm{FN}}_{{\mathrm{i}}} }}$$

8 $${\mathrm{Macro}}\;{\mathrm{Specificity}} = \frac{1}{{\mathrm{N}}}\mathop \sum \limits_{{{\mathrm{i}} = 1}}^{{\mathrm{N}}} \frac{{{\mathrm{TN}}_{{\mathrm{i}}} }}{{{\mathrm{TN}}_{{\mathrm{i}}} + {\mathrm{FP}}_{{\mathrm{i}}} }}$$

9 $${\text{ROC }} = { }\frac{1}{{\mathrm{N}}}\mathop \int \limits_{0}^{1} {\mathrm{TPR}}\left( {{\mathrm{FPR}}} \right){\mathrm{dFPR}}$$

Area under the ROC curve, where TPR = Sensitivity and

10 $${\mathrm{FPR}} = \frac{{{\mathrm{FP}}}}{{{\mathrm{FP}} + {\mathrm{TN}}}}$$

Log Loss (Binary Cross Entropy)

11 $${\text{Log Loss }} = { } - { }\frac{1}{{\mathrm{N}}}\mathop \sum \limits_{{{\mathrm{i}} = 1}}^{{\mathrm{N}}} \left[ {{\mathrm{y}}_{{\mathrm{i}}} \log ({\mathrm{p}}_{{\mathrm{i}}} ) + (1 - {\mathrm{y}}_{{\mathrm{i}}} )\log (1 - {\mathrm{p}}_{{\mathrm{i}}} )} \right]$$

where *y*_*i*_ true label (0 or 1). *p*_*i*_: predicted probability. *N*: total number of samples. TP: True Positives are the model accurate predictions. FP: False Positives are errors where the model outputs YES when it ought to be NO. TN: True Negatives are accurate model rejections. FN: False Negatives are errors that occur when the model responds with NO when it ought to respond with YES.

## Experimental results and analysis

The experimental setup consists of 10,000 patches, input to ResNet50, DenseNet201, EfficientNetB0, and ConvNeXt_Tiny for training and classification. 5000 benign and 5000 carcinoma samples are split into training, validation, and test datasets by two steps ensuring a balanced binary classification. First, 75% (7500 images) is used for training split into 3750 benign and 3750 carcinoma images. The remaining 25% (2500 images) is equally split into validation and test sets for both classes.

### Overall performance metrics

This section compares the performance of ResNet50, DenseNet201, EfficientNetB0, and ConvNeXt_Tiny. The evaluation is based on standard classification metrics, including accuracy, balanced accuracy, MCC, and Jaccard Index, as presented in Table 3. ResNet50 achieved an accuracy of 71.52% with an MCC of 0.4290, indicating its limited ability to distinguish between benign and malignant lesions in the dataset. The relatively low Jaccard Index (0.5558) further reflects its struggle to produce sufficient overlap between predicted and actual labels. DenseNet201 demonstrated improved performance, achieving an accuracy of 86.08% and an MCC of 0.7217. Its densely connected architecture likely facilitates feature propagation and mitigates the vanishing gradient problem, as reflected by a higher Jaccard Index of 0.7556. EfficientNetB0 significantly outperformed all other models across all evaluated metrics, attaining an accuracy of 97.6% and the highest MCC of 0.9519, indicating robust and reliable classification. Its compound scaling strategy, which balances network depth, width, and resolution, contributes to its superior performance, with a Jaccard Index of 0.9531, indicating minimal overlap error. ConvNeXt_Tiny achieved an accuracy of 95.92% and an MCC of 0.9184, placing it marginally behind EfficientNetB0. Despite being a relatively new model inspired by vision transformers while retaining CNN efficiency, ConvNeXt_Tiny demonstrates excellent performance, with a Jaccard Index of 0.9216, showing minimal overlap error.

**Table 3:** Overall performance metrics.

CNN models	Accuracy	Balanced Acc.	MCC	Jaccard Index
ResNet50	0.7152	0.7141	0.4290	0.5558
DenseNet201	0.8608	0.8608	0.7217	0.7556
EfficientNetB0	0.9760	0.9760	0.9519	0.9531
ConvNeXt_Tiny	0.9592	0.9592	0.9184	0.9216

#### Model accuracy curve

**Fig. 3:**
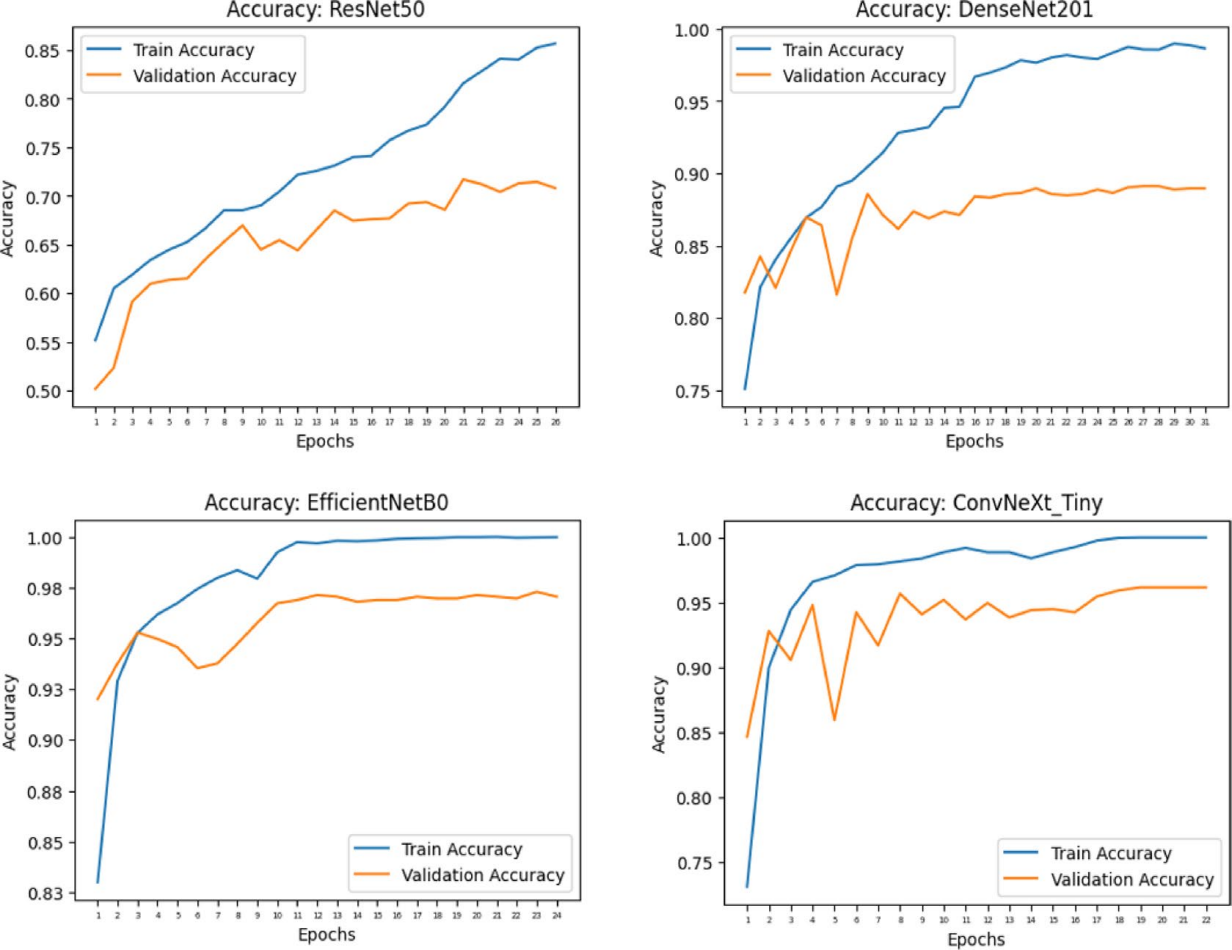
Model accuracy curve of ResNet50, DenseNet201, EfficientNetB0, & ConvNeXt_Tiny.

To evaluate the learning dynamics of each model, we analyzed the training and validation accuracy trajectories across epochs. The models ResNet50, DenseNet201, EfficientNetB0, and ConvNeXt_Tiny exhibit distinct behaviors in convergence rate, overfitting tendencies, and performance stability, as illustrated in Fig. 3. ResNet50 shows slow and gradual convergence over 26 epochs, with a persistent gap between training and validation accuracy. Validation accuracy plateaus around 0.70, while training accuracy continues to rise after epoch 10, indicating limited feature extraction and potential underfitting. DenseNet201 demonstrates moderately fast convergence, with both training and validation accuracies improving rapidly during the first 10 epochs before stabilizing. It generalizes better than ResNet50, with validation accuracy saturating around 0.88 and minimal overfitting. Its enhanced gradient flow and feature reuse suggest potential for further fine-tuning. EfficientNetB0 exhibits very rapid convergence, achieving over 95% validation accuracy within the first five epochs. It shows excellent generalization, with minimal divergence between training and validation curves, both approaching near-perfect accuracies. Overfitting is virtually absent, and validation accuracy remains stable. This performance is attributed to its compound scaling strategy, which optimizes network depth, width, and resolution. ConvNeXt_Tiny also displays extremely rapid convergence, reaching near-perfect training accuracy by about seven epochs and stabilizing validation accuracy above 0.95 by epoch ten. Its generalization performance is comparable to EfficientNetB0, albeit with slight fluctuations in validation accuracy. Overall, ConvNeXt_Tiny offers strong performance alongside efficient deployment potential.

### Per class and macro averaged metrics

The performance of ResNet50, DenseNet201, EfficientNetB0, and ConvNeXt_Tiny was evaluated based on sensitivity and specificity. Per-class metrics and macro-averaged metrics are presented in Table 4. ResNet50 achieved sensitivities of 0.7415 for benign and 0.6867 for carcinoma, with corresponding specificities of 0.7194 and 0.7103. This results in a macro sensitivity of 0.7141 and a macro specificity of 0.7149. DenseNet201 showed improved performance, with sensitivities of 0.8706 (benign) and 0.8510 (carcinoma), and specificities of 0.8542 and 0.8676, respectively, yielding macro sensitivity and specificity scores of 0.8608 and 0.8609. EfficientNetB0 outperformed both models, achieving sensitivities of 0.9753 and 0.9767, and specificities of 0.9784 and 0.9735, resulting in high macro sensitivity and specificity values of 0.9760 and 0.9759. ConvNeXt_Tiny also demonstrated strong performance, with sensitivities of 0.9551 and 0.9633, specificities of 0.9628 and 0.9556, and balanced macro scores of 0.9592 for both sensitivity and specificity.

**Table 4:** Per class and macro averaged metrics.

CNN Models	Sensitivity per class		Specificity per class		Macro sensitivity (recall)	Macro specificity
	Benign	Carcinoma	Benign	Carcinoma		
ResNet50	0.7415	0.6867	0.7194	0.7103	0.7141	0.7149
DenseNet201	0.8706	0.8510	0.8542	0.8676	0.8608	0.8609
EfficientNetB0	0.9753	0.9767	0.9784	0.9735	0.9760	0.9759
ConvNeXt_Tiny	0.9551	0.9633	0.9628	0.9556	0.9592	0.9592

#### Precision recall (PR) curve

**Fig. 4:**
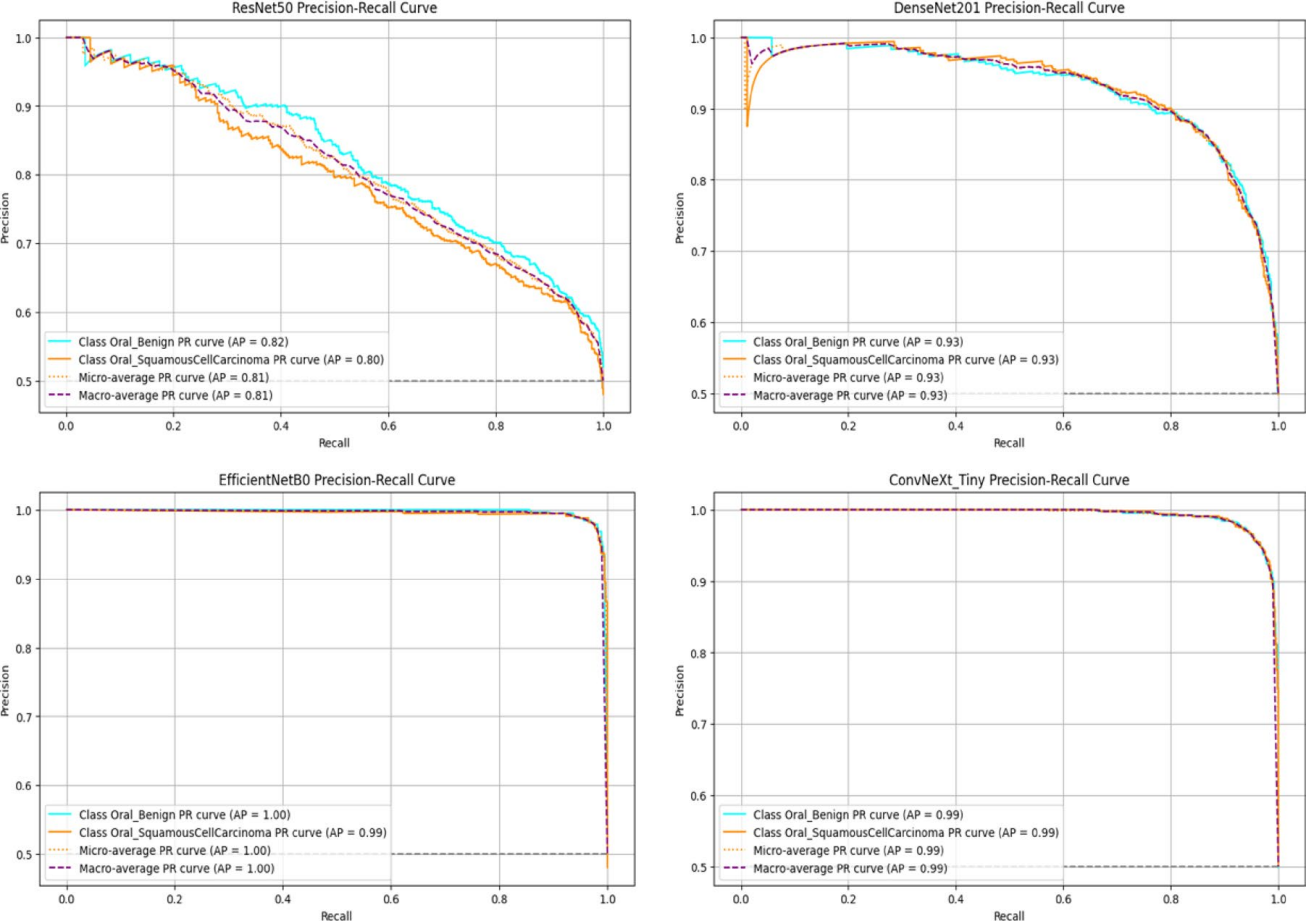
Precision recall curve of ResNet50, DenseNet201, EfficientNetB0, & ConvNeXt_Tiny.

Figure 4 presents a comparative analysis of ResNet50, DenseNet201, EfficientNetB0, and ConvNeXt_Tiny based on their Precision-Recall (PR) curves.

ResNet50 showed steadily increasing accuracy during training, reaching 85.6% on the training set and 71.6% on validation, indicating underfitting. The average precision (AP) score was 0.81, with low sensitivity and moderate specificity, suggesting limited class separation and unsuitability for precise carcinoma detection. DenseNet201’s accuracy plateaued around epoch 25, reaching 99% on training and 88% on validation, demonstrating better convergence than ResNet50. It exhibited slight overfitting and achieved an AP score of 0.93, with balanced sensitivity and specificity across both classes. EfficientNetB0 demonstrated rapid convergence by epoch 5, achieving 100% training accuracy and 98% validation accuracy. Its stable PR curves confirm reliability, with outstanding AP score, sensitivity, and specificity. ConvNeXt_Tiny achieved 100% training accuracy and 96% validation accuracy by epoch 15, showing fast, stable convergence and consistent performance. With AP scores near 0.99, it offers strong sensitivity and specificity, providing a lighter yet reliable alternative to EfficientNetB0 for real-time applications.

### Probability and threshold based metrics

ResNet50, DenseNet201, EfficientNetB0, and ConvNeXt_Tiny were evaluated using threshold-independent (ROC) and threshold-dependent (Log Loss) metrics, as shown in Table 5. EfficientNetB0 achieved the highest ROC of 0.9963 and the lowest log loss of 0.1003, indicating superior discrimination and calibration. ConvNeXt_Tiny also performed exceptionally well, with an ROC of 0.9932 and a low log loss of 0.1946.

**Table 5:** Probability and threshold based metrics.

Model	ROC score	Log loss
ResNet50	0.8077	0.5651
DenseNet201	0.9329	0.3856
EfficientNetB0	0.9963	0.1003
ConvNeXt_Tiny	0.9932	0.1946

DenseNet201 demonstrates strong performance with an ROC of 0.9329 and a Log Loss of 0.3856, outperforming ResNet50, which has an ROC of 0.8077 and a Log Loss of 0.5651. ResNet50 exhibits lower predictive power and less reliable probability estimates.

#### ROC curve

**Fig. 5:**
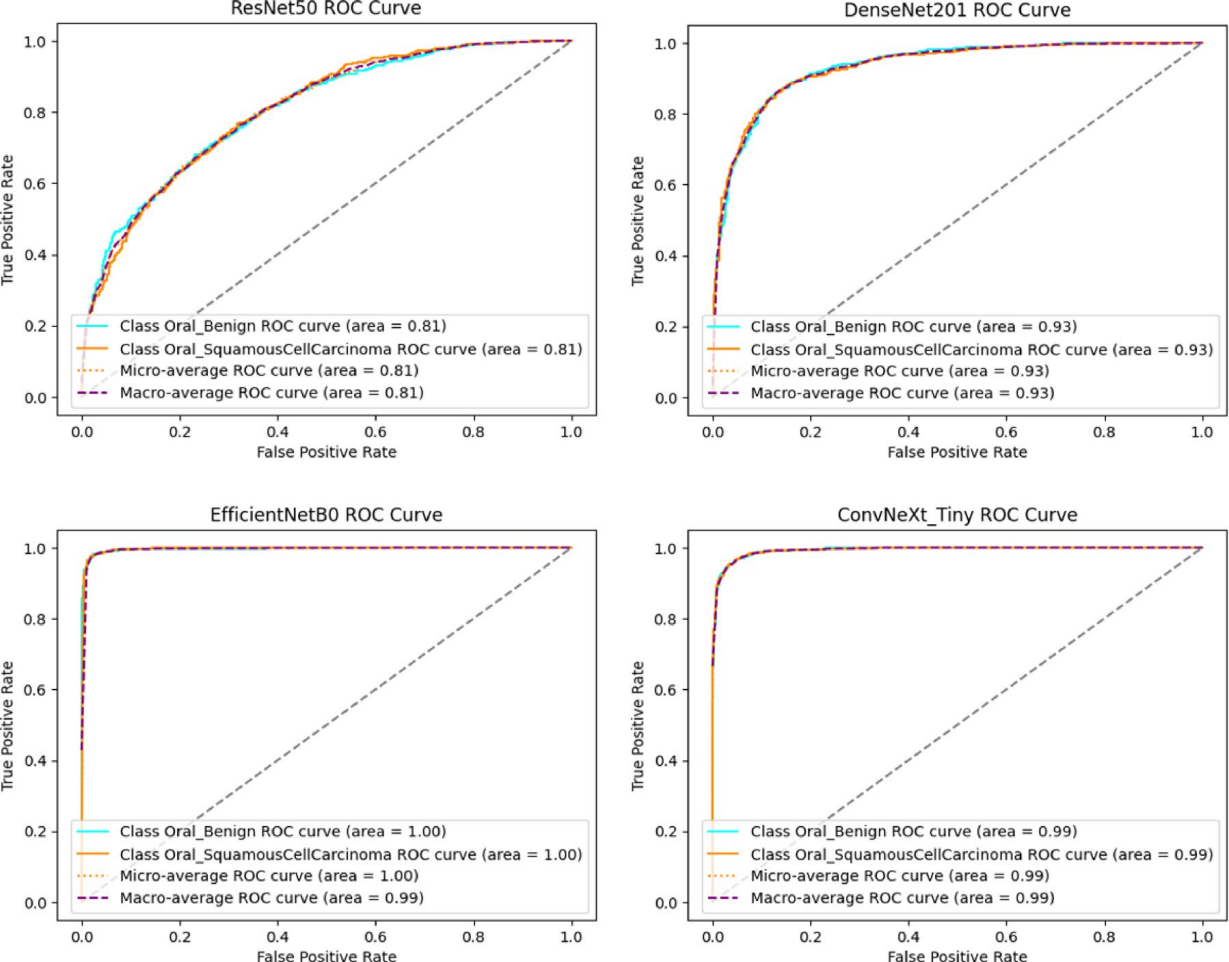
Receiver operating characteristic (ROC) Curve of ResNet50, DenseNet201, EfficientNetB0, & ConvNeXt_Tiny.

The ROC curves shown in Fig. 5 depict the true positive rate versus the false positive rate for each model, providing a measure of their discriminative power across all classification thresholds. These visual curves corroborate the quantitative results.

The ROC curve of ResNet50 displays a relatively flattened trajectory compared to the other models, highlighting its weaker discriminative performance. DenseNet201, EfficientNetB0, and ConvNeXt_Tiny produce steep curves that approach the top-left corner of the graph, representing ideal behavior for binary classifiers. The curves for EfficientNetB0 and ConvNeXt_Tiny nearly overlap the top-left boundary, indicating excellent sensitivity and specificity across all thresholds. DenseNet201 follows a similar path with minor deviations, reflecting slightly inferior yet strong classification performance.

#### Training and validation loss curves

**Fig. 6:**
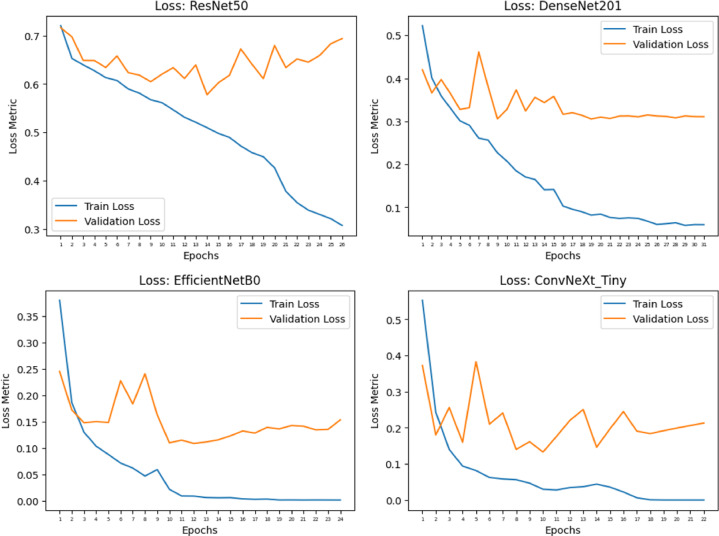
Training and validation loss curves of ResNet50, DenseNet201, EfficientNetB0, & ConvNeXt_Tiny.

As shown in Fig. 6, the training loss of ResNet50 consistently decreases, while the validation loss fluctuates at a higher value toward the end, indicating significant overfitting. For DenseNet201, both training and validation losses decrease initially, with the validation loss stabilizing above the training loss. The narrower gap between training and validation losses compared to ResNet50 suggests improved generalization and reduced overfitting.

EfficientNetB0 achieves rapid and substantial reductions in both training and validation loss, with both curves flattening at low values. The small gap between them and the low absolute loss values indicate an excellent fit. In ConvNeXt_Tiny, the training loss drops quickly and remains low, while the validation loss fluctuates but stays relatively low. This behavior suggests good generalization, with some instability in validation loss likely due to data variability or model sensitivity.

### Confusion matrix

**Fig. 7:**
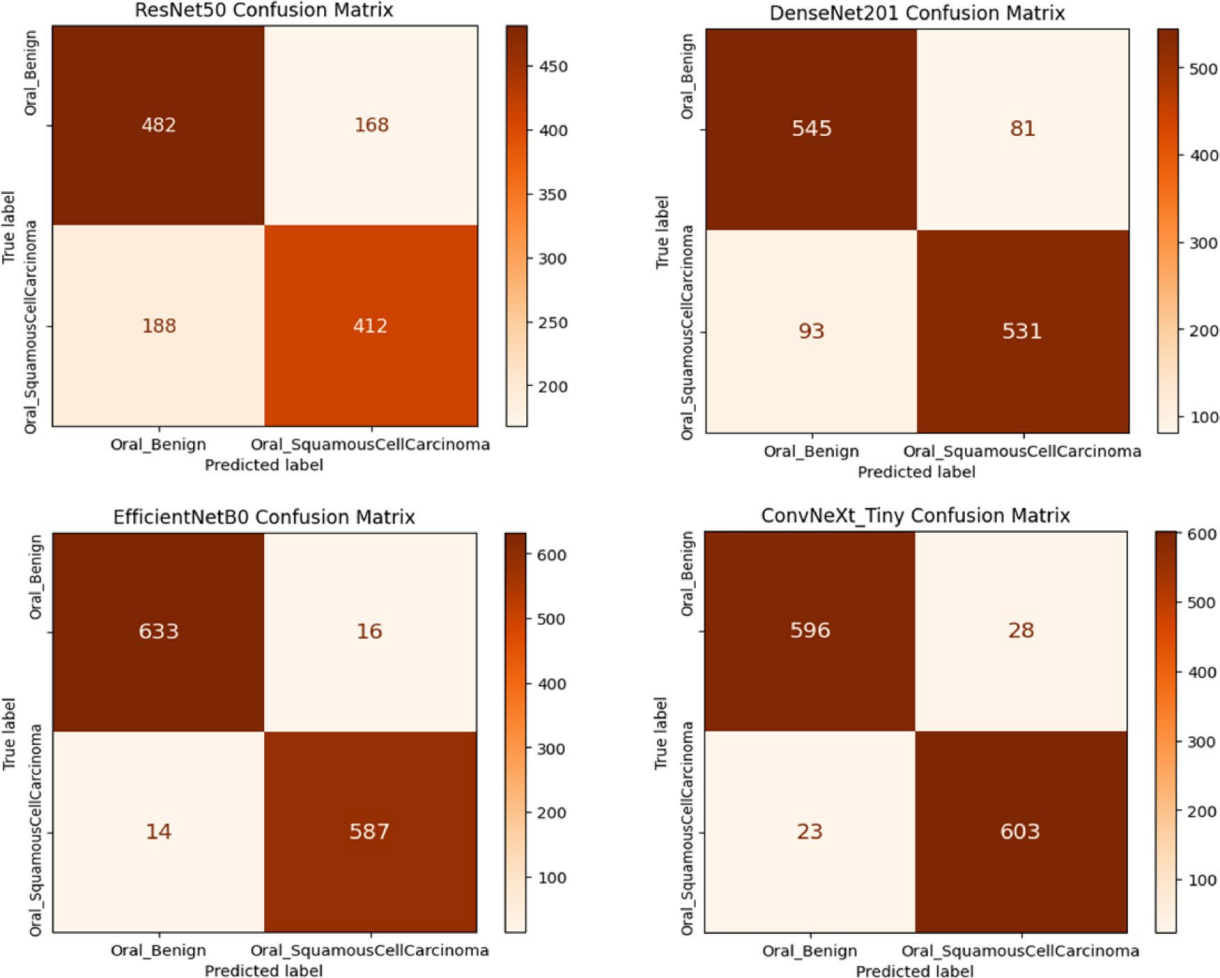
Confusion matrix of ResNet50, DenseNet201, EfficientNetB0, & ConvNeXt_Tiny.

The confusion matrices for ResNet50, DenseNet201, EfficientNetB0, and ConvNeXt_Tiny are shown in Fig. 7. ResNet50 exhibits the weakest performance, with relatively high misclassification rates: 168 benign cases are incorrectly identified as carcinoma, and 188 carcinoma cases are misclassified as benign. DenseNet201 shows significant improvement, reducing misclassifications to 81 benign and 93 carcinoma cases, indicating balanced and accurate performance. EfficientNetB0 stands out as the best-performing model, correctly identifying 633 out of 649 benign cases and 587 out of 601 carcinoma cases, with only 30 total misclassifications. ConvNeXt_Tiny also demonstrates strong performance, slightly trailing EfficientNetB0, with 28 benign and 23 carcinoma cases misclassified.

## Comparative analysis

**Fig. 8:**
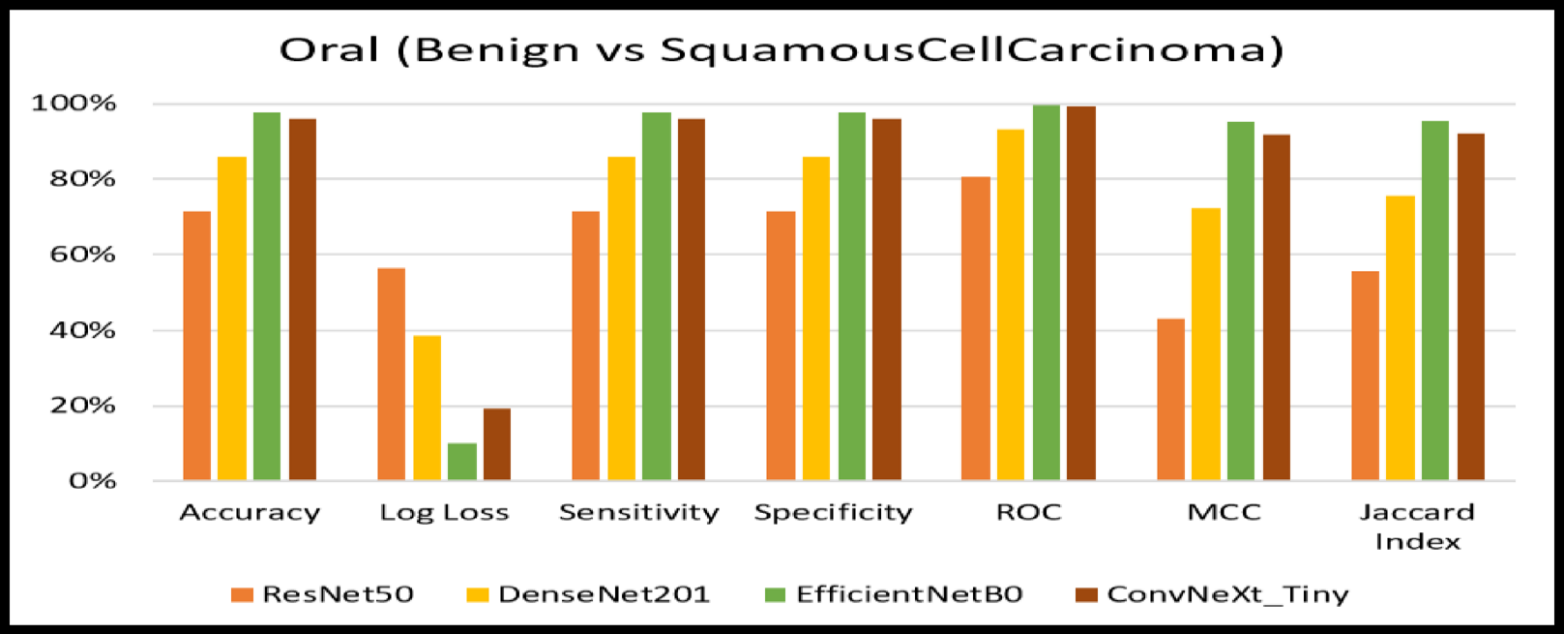
Comparative Analysis of ResNet50, DenseNet201, EfficientNetB0, & ConvNeXt_Tiny.

The comparative analysis of all four models is presented in Fig. 8. The ground truth in this study was expert pathological annotation for the oral histopathology images. Each image was defined by authenticated oral pathologists after microscopic assessment of tissue structure and cell structure. Lesions were classified into two broad diagnostic categories—benign and carcinoma. These diagnostic labels, put under the microscope by experts, were used as the ground truth for training, validating, and testing the model. All four CNN models were evaluated for the degree of their accuracy and reliability against these expert-determined labels. (less confidence to identify oral lesions) as compared to the other models. ResNet50, a widely adopted model known for its residual learning capabilities, demonstrated the weakest performance among them. Its overall metrics indicate weaker agreement with the ground truth labels, thereby exhibiting lower accuracy and lower reliability. The macro-averaged sensitivity and specificity further highlight its limitations in this clinical context. While ResNet50 provided a reasonable baseline, its relatively high log loss suggests less confident probability estimates. Although its depth enables hierarchical feature extraction, it lacks the advanced scaling and connectivity mechanisms found in newer models. DenseNet201 showed considerable improvement, leveraging its densely connected architecture to promote feature reuse and improved gradient flow. It achieved higher class-specific sensitivities and specificities compared to ResNet50, with an ROC score reflecting greater discriminative power and robustness. Despite outperforming ResNet50, DenseNet201 still lags behind the more modern models in terms of confidence and generalization.

EfficientNetB0 represents a transformative leap in classification performance. Designed for optimal scaling with fewer parameters, it excelled across nearly all evaluation metrics. Its class-wise sensitivity and specificity reached very high levels, and the MCC reflects a highly reliable classifier. A low log loss of just 0.1003 further indicates strong model calibration, which is crucial for medical image analysis. ConvNeXt_Tiny, a recent model inspired by vision transformers but implemented with convolutional architectures, also delivered outstanding results. Achieving a high accuracy of 95.92%, it closely trailed EfficientNetB0 while significantly outperforming ResNet50 and DenseNet201. Its Jaccard Index and balanced accuracy confirm strong performance across class distributions. Although ConvNeXt_Tiny has a slightly higher log loss than EfficientNetB0, its predictive power remains highly impressive. By integrating transformer design principles—such as patch-based processing and larger receptive fields—within a CNN framework, ConvNeXt_Tiny offers computational efficiency and enhanced feature representation, key factors for fine-grained lesion classification.

## Discussion

Our study differs from previous research in several ways. Previous studies predominantly used conventional convolutional neural networks (CNN). They utilised VGG16, InceptionV3, and ResNet50, or hybrid frameworks combining CNN features with handcrafted descriptors like GLCM, LBP, and HOG as shown in Table 6. No doubt these researches achieved promising accuracies, but suffered from limited generalization, smaller datasets, in contrast, this study explored four diverse and advanced architectures—ResNet50 (residual learning), DenseNet201 (dense connectivity), EfficientNetB0 (compound scaling), and ConvNeXt_Tiny (transformer-based convolutions)—for binary classification of 10,000 histopathological images of oral tissue.

**Table 6:** Comparative analysis with works.

References	Details
Rahman et al.^18^	Dataset: 4946 Images Model: AlexNet Maximum Accuracy: 90.06% Key Highlights: Early use of AlexNet for histopathology image classification using transfer learning
Panigrahi et al.^19^	Dataset: 52,189 Patches Model: VGG16, VGG19, InceptionV3, ResNet50, MobileNet, Baseline CNN Maximum Accuracy: 96.60% Key Highlights: Compared several pre-trained CNN models; established baseline CNN performance on large patch-level dataset
Deo et al.^22^	Dataset: 696 Images Model: 2D-EWT, ResNet50, DenseNet201 Maximum Accuracy: 92.00% Key Highlights: Introduced ensemble approach combining multiple CNN architectures for improved accuracy
Ahmad et al.^23^	Dataset: 5192 Images Model: GLCM, LBP, HOG, Xception, InceptionV3, NASNetLarge, DenseNet201, SVM Maximum Accuracy: 97.00% Key Highlights: Combined texture-based features with CNN deep features using hybrid (SVM + CNN) framework
Das et al.^24,21^	Dataset: 126 Images Model: Gabor Filters, Random Forest Maximum Accuracy: 96.88% Key Highlights: Applied handcrafted texture filters with classical machine learning classifier
Maia et al.^10^	Dataset: 3763 Patches Model: ResNet50, MobileNet, DenseNet121, VGG16, coat_lite_small, pit_s_distilled_224 Maximum Accuracy: 91.91% Key Highlights: Compared CNN and ViT-based models on histopathology patches; demonstrated consistent CNN performance
Silva et al.^26^	Dataset: 66 Images Model: Polynomial Classifier Maximum Accuracy: 92.4% Key Highlights: Used a polynomial-based statistical classifier for histopathology image classification on a small dataset
Proposed study	Dataset: 10,000 Images Model: ResNet50, DenseNet201, EfficientNetB0, ConvNeXt_Tiny Maximum Accuracy: 97.60% Key Highlights: Uses the largest dataset among compared studies. Performs a comparative evaluation across four deep architectures (residual, dense, compound-scaled, transformer-based). Introduces ConvNeXt_Tiny, bridging CNN and transformer designs. Employs comprehensive metrics (Accuracy, Balanced Accuracy, MCC, Jaccard Index, ROC, Log Loss). Demonstrates robust generalization and superior balanced performance

The results highlight the clear advantage of scaling efficiency and transformer-inspired models. Additionally, this study employs comprehensive evaluation metrics, including Balanced Accuracy, MCC, Jaccard Index, Sensitivity, and Specificity, providing a fairer and more robust assessment than accuracy alone. These findings demonstrate that next-generation CNNs can better capture complex histopathological features, ensure greater diagnostic reliability and pave the way for real-world clinical integration in oral cancer detection.

### Practical implications

From a clinical standpoint, models with high specificity are crucial to minimize false positives that could lead to unnecessary biopsies or treatments. Equally important is high sensitivity to ensure early detection of malignant cases. Both EfficientNetB0 and ConvNeXt_Tiny meet these criteria, providing reliable classification with high accuracy and well-calibrated confidence. These models are not only theoretically efficient but also practically suited for deployment in diagnostic tools and mobile health platforms. Their ability to detect subtle features distinguishing carcinoma from benign tissue demonstrates strong generalizability to real-world clinical workflows, supporting early diagnosis. The superior performance of EfficientNetB0 highlights the significance of architectural scaling and optimization, especially in medical image classification where computational resources and labelled data may be limited. Meanwhile, ConvNeXt_Tiny illustrates how integrating transformer-inspired elements into CNN can yield models that are both powerful and efficient, effectively combining the strengths of both architectures. Despite these advancements, certain limitations must be acknowledged. While the tested models demonstrate high performance on the 10,000-image dataset, validation across multi-center datasets is necessary to ensure robustness under real-world conditions. This would incorporate a broader demographic and diverse histopathological image preparation protocols. Model interpretability remains an ongoing challenge; black-box predictions can be difficult for clinicians to trust without a clear rationale. The integration of explainable AI techniques, such as attention heatmaps or saliency mapping, could significantly enhance the clinical acceptability of these models.

## Conclusion and future work

This study presents a comprehensive comparative evaluation of four CNN models for OSCC classification using a high-quality dataset of 10,000 histopathological images. Among the evaluated models, EfficientNetB0 demonstrates the best overall performance, achieving strong scores across accuracy, sensitivity, specificity, ROC, and log loss. Its ability to accurately distinguish between benign and carcinoma cases with minimal error makes it the most suitable candidate for deployment in automated diagnostic systems. ConvNeXt_Tiny closely follows, offering a practical balance between computational efficiency and high classification performance. DenseNet201, while still performing well, lags slightly due to higher resource demands and less optimal confidence calibration. ResNet50, although historically popular, shows clear limitations in handling complex histopathological data, making it less appropriate for critical diagnostic use. These findings advocate for the integration of EfficientNetB0 and ConvNeXt_Tiny into clinical workflows for OSCC screening and diagnosis. Future work should focus on improving interpretability, enhancing domain generalization, and developing deployment strategies suitable for real-world pathology laboratories. Incorporating multimodal data (e.g., genomics, radiomics) alongside histopathological images could further improve diagnostic accuracy and clinical trust. Ensemble learning approaches combining EfficientNet and ConvNeXt variants may also boost performance. Investigating lightweight versions of these models optimized for deployment on edge devices could enable real-time inference in low-resource settings. Additionally, domain adaptation strategies should be explored to ensure consistent performance across histopathological images obtained from different staining protocols or imaging devices. Finally, extending the classification from binary to multi-class would better align the models with clinical diagnostic pathways and provide deeper insights.

## Data Availability

The datasets generated and/or analysed during the current study are publicly available on Zenodo under a CC-BY 4.0 license. Oral benign and carcinoma histopathological images stained with haematoxylin and eosin (H&E) were collected from the ORCHID dataset, which served as the primary input for classification. The datasets can be directly accessed at: https://zenodo.org/records/12636426 and https://zenodo.org/records/12646943).

## Code availability

10.5281/zenodo.18903644.
